# Parental Knowledge and Acceptance of HPV Vaccine in Rabigh’s School, Saudi Arabia

**DOI:** 10.5334/aogh.4866

**Published:** 2026-02-02

**Authors:** Raneem Alghanmi, Eman Alkhalawi, Roaa Albeladi, Shahad Albeladi, Munirah Alghamdi, Abdlkareem Fayoumi, Rawan Nassif

**Affiliations:** 1 Faculty of Medicine, King Abdulaziz University, Rabigh, Saudi Arabia; 2 Department of Family and Community Medicine, Faculty of Medicine, King Abdulaziz University, Rabigh, Saudi Arabia; 3 Department of Obstetrics and Gynecology, Faculty of Medicine, King Abdulaziz University, Rabigh, Saudi Arabia

**Keywords:** HPV vaccine, parental knowledge, vaccine acceptance, school-based vaccination, Saudi Arabia, cervical cancer prevention

## Abstract

*Background:* Human papillomavirus (HPV) is a common sexually transmitted infection associated with cervical cancer. Since 2008, HPV vaccines have been available in Saudi Arabia, and in 2022, a nationwide school-based vaccination program was launched to improve coverage and reduce HPV-related diseases.

*Objectives:* This study evaluated parental knowledge and acceptance of the HPV vaccine during a school-based vaccination campaign in Rabigh, Saudi Arabia. It also examined associations between demographic factors, parental knowledge, and vaccine acceptance.

*Methods:* A cross-sectional study involved 261 guardians of girls attending intermediate schools in Rabigh during the academic years 2022/2023, 2023/2024, and 2024/2025. Data were collected through an online questionnaire assessing HPV vaccine knowledge, acceptance, and influencing factors. Chi-square tests were used for analysis.

*Findings:* Vaccine acceptance increased with guardians’ educational qualification, from 46.7% among those with primary or middle school to 89.3% among those with higher education (*P* = 0.007). Acceptance also increased with family income, from 45.2% (income < 5000 SR) to 77.7% (income ≥ 10,000 SR) (*P* = 0.002), and was higher when information was obtained from health practitioners (90.4%) or the Internet (80.4%) compared to relatives or social media (47%) (*P* < 0.001). Respondents with a knowledge score ≥80% were more likely to accept the vaccine (84.2% versus 67.3%, *P* = 0.04). Recommendations from the Ministry of Health (MOH) or physicians were key motivators for vaccination. Concerns about vaccine safety were the primary reason for refusal (48.1%). Among non-accepting parents, 59.5% reported that more information on benefits and safety would encourage acceptance, while 22.8% remained unwilling to vaccinate.

*Conclusion:* Parental knowledge, education, and income significantly influenced HPV vaccine acceptance. Parents informed by healthcare professionals or the Internet were more likely to vaccinate their daughters. Targeted efforts raising awareness of vaccine benefits and safety from trusted sources like physicians and the MOH are essential.

## Introduction

Human papillomavirus (HPV) is one of the most common sexually transmitted infections worldwide, affecting approximately 12% of women with normal cytology [1]. It primarily infects the skin and mucous membranes [2]. Most sexually active individuals, irrespective of gender, will contract HPV at some point, often in early adulthood, shortly after the initiation of sexual activity [3]. In women, HPV prevalence peaks under the age of 25 and again after 45, influenced by immune and behavioral factors [1, 4]. While many HPV infections resolve spontaneously, the virus can cause genital warts and cancers, including cervical cancer [5].

Cervical cancer is largely preventable through early detection and treatment of precancerous lesions via Pap tests and HPV vaccination [6]. Therefore, and recognizing its global burden, the World Health Organization (WHO) launched in 2018 its strategy to eliminate cervical cancer, aiming for an incidence rate below 4 per 10,000 women in all countries [3, 7]. WHO recommends vaccinating girls aged 9–14, prior to the onset of sexual activity, to maximize vaccine benefits. This age group is the primary target for HPV vaccination programs [1].

The US FDA has authorized three vaccines: Gardasil (quadrivalent), Cervarix (bivalent), and Gardasil 9 (9-valent), which all protect against HPV types 16 and 18. Additionally, Gardasil and Gardasil 9 protect against HPV types 6 and 11, which cause genital warts, while Gardasil 9 covers five more high-risk types associated with cancers [8]. Although three doses were initially considered optimal, recent evidence shows that one dose provides strong efficacy and long-term immunity [9].

In 2008, Saudi Arabia approved Cervarix and Gardasil for girls aged 9 and older [10, 11]. The Ministry of Health recommends two doses for girls aged 9–14, given 6–12 months apart. For females aged 15 and older, a three-dose protocol is followed [12]. In March 2022, a school-based HPV vaccination campaign targeting seventh-grade girls was launched in collaboration with the Ministry of Education. By September 2022, the vaccine became available at primary healthcare centers for females aged 9–18, upon request [8].

Despite these efforts, cultural barriers and misconceptions hinder vaccine acceptance in Saudi Arabia. A study in Jazan Province highlighted these challenges, with almost a third of adults in the general population opposing the vaccine [13]. Globally, HPV vaccines initially faced mixed public and governmental responses due to their focus on preventing a sexually transmitted disease [14]. However, extensive awareness campaigns in developed countries have significantly increased vaccine uptake, creating optimism about eliminating cervical cancer. While challenges remain in achieving universal HPV vaccination, projections suggest the potential eradication of HPV-associated cancers in the 21st century [14].

Studies in Tabuk and Jazan cities revealed inadequate understanding and awareness of HPV vaccination in Saudi Arabia [13, 15]. Educational intervention has been shown to increase knowledge, which in turn has improved acceptance of the HPV vaccine among secondary school girls [16, 17].

This study evaluates the knowledge and willingness of parents in Rabigh, Saudi Arabia, to accept the HPV vaccine for their school-age daughters and explores associations between demographic variables, parental knowledge, and vaccine acceptance.

## Methodology

### Study design and sample size

This study employed an analytic cross-sectional design. The study population included parents of girls enrolled in middle school during the HPV vaccination campaign in the academic years 2022/2023, 2023/2024, and 2024/2025. Parents who were not residents of Rabigh or whose daughters did not attend middle school during the campaign were excluded.

A sample size of 261 eligible participants was determined, assuming a 95% confidence level, a 5% margin of error, a response distribution of 70% vaccine acceptance according to previous studies and a population size of 810 (approximate number of female students in Rabigh middle schools). The sample size was calculated in Rao software.

### Data collection

Data collection was conducted between December 1 and December 20, 2024. Following approval from the school administration, a self-administered online questionnaire in Arabic was electronically distributed to all parents or guardians of students attending girls’ middle schools in Rabigh through school authorities.

The questionnaire was self-constructed based on questionnaires used in previous studies [15, 18], and was translated into Arabic. Its content was reviewed and validated by three experts. The final version included 30 questions, divided into three sections:

1. Demographic data (nine questions): Included participants’ age, marital status, and additional details about their daughter.
2. Knowledge of the HPV vaccine (ten questions): A score of 80% or higher was qualified as good knowledge.
3. Vaccine acceptance (nine questions): Assessed parental acceptance, including reasons for consenting or refusing vaccination. Parental agreement for their daughters to receive the vaccine was considered a measure of acceptance.

Internal consistency was examined using Cronbach’s alpha (equivalent to the Kuder–Richardson 20 coefficient for dichotomous items), which yielded a coefficient of 0.65 for the overall scale, indicating moderate internal consistency. Given that Cronbach’s alpha is sensitive to test length and tends to be lower for short scales, this level of reliability was considered acceptable for the exploratory, group-level analyses in the present study.

Ethical approval was obtained from the Research Ethics Committee of King Abdulaziz University. Jeddah (Reference No: 24016). Informed consent was obtained from all participants before they proceeded with the questionnaire.

### Data analysis

Data were analyzed using IBM SPSS Statistics (version 20) [19]. Chi-square test was performed to assess the association between both parental demographics and parental knowledge (good knowledge was determined as 80% or higher), and the decision to vaccinate their daughters. An independent *t*-test was used to assess the association between age and vaccine acceptance.

## Results

Table 1 shows the characteristics and responses of 261 parents who consented to take part and completed the survey (92.5%). The mean age of the participants was 43.8 (SD = 8.4). The majority of respondents were mothers (63.6%), Saudis (92.7%), married (91.2%), university graduates (62.8%), employed (48.3%), and had a family income of more than 10,000 SR (46.7%). Most participants had one daughter in middle school during the period under study (80.8%), and all their daughters completed the basic vaccination schedule/immunization card. The HPV vaccine was offered to 40.6% during 1446 AH (academic year 2024/2025).

**Table 1:** Demographic characteristics, knowledge score questions, and decision-making factors (*n* = 261).

DEMOGRAPHIC DATA			
		**Frequency**	**Percent (%)**
**Age (year)**	Mean, SD	43.8	8.4
**Relationship of the participant with the student**	Mother	166	63.6
	Father	85	32.6
	Other	10	3.8
**Nationality**	Saudi	242	92.7
	Non-Saudi	19	7.3
**Marital status**	Married	238	91.2
	Divorced	16	6.1
	Widow	7	2.7
**Educational level**	Primary school/middle school	15	5.7
	High school	54	20.7
	University	164	62.8
	Higher education	28	10.7
**Employment**	Employee	126	48.3
	Not employed	87	33.3
	Freelancer	30	11.5
	Retired	18	6.9
**Family income**	Less than 5000 SR	31	11.9
	5000–10,000 SR	108	41.4
	More than 10,000 SR	122	46.7
**Number of daughters**	One	211	80.8
	Two	41	15.7
	Three	9	3.4
**Has the student completed the basic vaccination schedule/immunization card?**	Yes	261	100.0
	No	0	0.0
**In which year was your daughter offered the HPV vaccine?**	1444 (2022/2023)	100	38.3
	1445 (2023/2024)	55	21.1
	1446 (2024/2025)	106	40.6
**Knowledge scoring questions (correctly answered)**			
		**Frequency**	**Percent (%)**
**Have you ever heard of cervical cancer?**	Yes	212	81.2
**Have you ever heard of HPV before the school vaccination campaign?**	Yes	110	42.1
**HPV infection causes burning sensation during urination**	False	39	14.9
**HPV infection causes genital warts**	True	97	37.2
**HPV infection affects fertility**	False	11	4.2
**HPV infection is sexually transmitted**	True	139	53.3
**HPV infects females only**	False	94	36.0
**In your opinion, is there a relationship between HPV and cervical cancer?**	Yes/maybe	256	98.5
**Do you think the HPV vaccine helps prevent cervical cancer**	Yes/maybe	251	96.2
**Do you know who the vaccine target group is?**	Girls and women aged 9–25 years	203	77.8
**Decision-making factors**			
		**Frequency**	**Percent (%)**
**How long did it take you to decide after receiving the vaccination consent paper?**	One day	153	58.6
	2–5 days	90	34.5
	More than 5 days	18	6.9
**What sources did you turn to for additional information about vaccination?**	Family and relatives	19	7.3
	Internet	112	42.9
	Social media	36	13.8
	Health practitioners	52	19.9
	No additional information was needed	42	16.1
**Who was the person involved in making the decision?**	Mother	57	21.8
	Father	31	11.9
	Both parents	151	57.9
	The student	22	8.4
**Among the following reasons, which is the most important reason that would encourage you to vaccinate your daughter/daughters?**	If a family member vaccinates his daughters	5	1.9
	If recommended by the Ministry of Health or a doctor	141	54.0
	Get more information about its benefits and safety	97	37.2
	I will not vaccinate my daughter(s)	18	6.9
**If you did not agree for your daughter to take the vaccination, what is the main reason that led you to make this decision? (*n* = 79)**	We prefer for our daughter to take it later	8	10.1
	We need more information	7	8.9
	Fear of side effects and complications	38	48.1
	We think our daughter doesn’t need it	7	8.9
	Vaccination is still new	19	24.1
**Knowledge score**	Good	38	14.6
	Poor	223	85.4

Most of the responding parents had heard of cervical cancer at the time of the survey (*n* = 212, 81.2%). But only 42.1% (*n* = 110) had heard of HPV prior to the school campaign. Knowledge about the signs and symptoms of HPV infection was poor: only 14.9% correctly disagreed that HPV causes painful urination, 37.2% agreed that HPV causes genital warts (*n* = 97, 37.2%), and only 4.3% agreed that HPV infection does not affect fertility (*n* = 11). Approximately half of the participants (*n* = 139, 53.3%) knew that HPV is a sexually transmitted infection, and 36% disagreed that it only affects females (*n* = 94). The majority of parents related HPV infection with cervical cancer (*n* = 256, 98.5%) and believed that the HPV vaccine can help prevent cervical cancer (*n* = 251, 96.3%). Furthermore, most parents correctly identified the targeted vaccination group as girls and women aged 9 to 25 years (*n* = 203, 77.8%). Overall, the mean knowledge score was 5.4 (SD = 2.0, range = 2–10), and only 14.6% of respondents were considered to have a good knowledge score.

Most parents took a decision regarding consenting to the vaccine within one day (*n* = 153, 58.6%). The primary source of information for many was the Internet, used by 42.9% (*n* = 112). Both parents participated in the decision in 57.9% of families. Additionally, parents mostly felt encouraged by recommendations from the MoH or doctors (*n* = 141, 54%). Among those who refused the vaccine, the most frequent reason for refusing vaccination was concerns about side effects and complications (*n* = 79, 48.1%).

**Figure 1:**
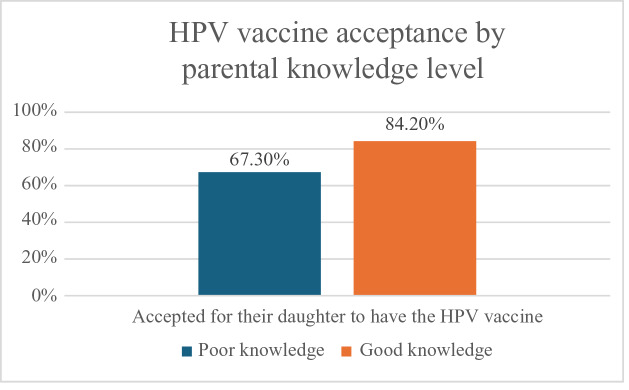
HPV vaccine acceptance by parental knowledge level.

Table 2 and Figure 1 demonstrate that vaccine acceptance was higher among those with good knowledge (84.2%) compared to those with poor knowledge (67.3%) (*P* = 0.04).

**Table 2:** Relationship between knowledge and HPV vaccine acceptance.

		ACCEPTED FOR THEIR DAUGHTER TO HAVE THE HPV VACCINE		CHI-SQUARE VALUE (*P*-VALUE)
		NO (*N* = 79)	YES (*N* = 182)	
**Knowledge score**	Poor knowledge^a^	73 (32.7%)	150 (67.3%)	4.48 (0.04)
	Good knowledge^b^	6 (15.8%)	32 (84.2%)	

^a^Poor knowledge: a knowledge score lower than 80%.

^b^Good knowledge: a knowledge score of 80% or higher.

Table 3 shows HPV vaccine acceptance, measured by consenting to be vaccinated, was higher among girls whose fathers responded to the survey (74.1%), compared to those whose mothers responded (65.7%) (*P* = 0.04). Acceptance was slightly higher among non-Saudi parents (73.7%) than Saudis (69.4%) (*P* = 0.80) and among married guardians (71.0%) compared to divorced (68.8%) or widowed (28.6%) guardians (*P* = 0.06). The acceptance of the HPV vaccine consistently increased with educational attainment, ranging from 89.3% among guardians who had a higher education degree to 46.7% among those with primary or middle school education (*P* = 0.007). Likewise, the acceptance of the HPV vaccine in those who were employed or freelancers (74.6% and 73.3%, respectively) was higher than those who were not employed or retired (62.1% and 66.7%, respectively) (*P* = 0.243). Acceptance also increased with increasing family income. A total of 77% of parents with a monthly family income of more than 10,000 SR accepted the HPV vaccine compared to only 45.2% of those with a family income of less than 5000 SR (*P* = 0.002).

**Table 3:** Relationship between HPV vaccine acceptance and participant characteristics.

		ACCEPTED FOR THEIR DAUGHTER TO HAVE THE HPV VACCINE		
		NO (*N* = 79)	YES (*N* = 182)	
		**Mean ± SD**	**Mean ± SD**	***P*-value (independent *t*-test)**
**Age by year**		43.87 ± 8.29	43.70 ± 8.69	0.88
		**Frequency (%)**	**Frequency (%)**	***P*-value (chi-square tests)**
**Relationship of the participant with the student**	Mother	57 (34.3%)	109 (65.7%)	0.04
	Father	22 (25.9%)	63 (74.1%)	
	Other	0 (0.0%)	10 (100.0%)	
**Nationality**	Saudi	74 (30.6%)	168 (69.4%)	0.8
	Non-Saudi	5 (26.3%)	14 (73.7%)	
**Marital status**	Married	69 (29.0%)	169 (71.0%)	0.055
	Divorced	5 (31.3%)	11 (68.8%)	
	Widow	5 (71.4%)	2 (28.6%)	
**Educational level**	Primary school/middle school	8 (53.3%)	7 (46.7%)	0.007
	High school	22 (40.7%)	32 (59.3%)	
	University	46 (28.0%)	118 (72.0%)	
	Higher education	3 (10.7%)	25 (89.3%)	
**Employment**	Employee	32 (25.4%)	94 (74.6%)	0.251
	Not employed	33 (37.9%)	54 (62.1%)	
	Freelancer	8 (26.7%)	22 (73.3%)	
	Retired	6 (33.3%)	12 (66.7%)	
**Family income**	Less than 5000 SR	17 (54.8%)	14 (45.2%)	0.002
	5000–10,000 SR	34 (31.5%)	74 (68.5%)	
	More than 10,000 SR	28 (23.0%)	94 (77.0%)	

Table 4 shows the associations between decision-making factors and acceptance of the HPV vaccine. Parents who took more than five days to reach a decision were substantially less likely to accept the vaccine (27.8%, *P* < 0.001). Parents who sought additional information from healthcare practitioners showed the highest acceptance level (90.4%), followed by those who used the Internet (80.4%). In contrast, less than 50% of those who obtained extra information from relatives or social media or did not seek additional information at all accepted the vaccine (*P* < 0.001). Acceptance was higher when both parents were involved in decision-making (75.5%) compared to mothers, fathers, or daughters alone (*P* = 0.09). Furthermore, 92.2% of parents mentioned that the most important reason that would encourage them to vaccinate their daughters was recommendations from the health ministry or a doctor (*P* < 0.001). Also, 22.8% (*n* = 18) of those who did not accept the vaccine said they would not vaccinate their daughters.

**Table 4:** Relationship between acceptance and decision-making factors.

		ACCEPTANCE		*P*-VALUE OF CHI-SQUARE TEST
		NO (*N* = 79)	YES (*N* = 182)	
**How long did it take you to decide after receiving the vaccination consent paper?**	One day	46 (30.1%)	107 (69.9%)	<0.001
	2–5 days	20 (22.2%)	70 (77.8%)	
	More than 5 days	13 (72.2%)	5 (27.8%)	
**What sources did you turn to for additional information about vaccination?**	Family and relatives	10 (52.6%)	9 (47.4%)	<0.001
	Internet	22 (19.6%)	90 (80.4%)	
	Social media	19 (52.8%)	17 (47.2%)	
	Health practitioners	5 (9.6%)	47 (90.4%)	
	No additional information was needed	23 (54.8%)	19 (45.2%)	
**Who was the person involved in making the decision?**	Mother	20 (35.1%)	37 (64.9%)	0.09
	Father	12 (38.7%)	19 (61.3%)	
	Both parents	37 (24.5%)	114 (75.5%)	
	The student	10 (45.5%)	12 (54.5%)	
**Among the following reasons, which is the most important reason that would encourage you to vaccinate your daughter/daughters?**	If a family member vaccinates his daughters	3 (60.0%)	2 (40.0%)	<0.001
	If recommended by the Ministry of Health or a doctor	11 (7.8%)	130 (92.2%)	
	Get more information about its benefits and safety	47 (48.5%)	50 (51.5%)	
	I will not vaccinate my daughter(s)	18 (100.0%)	0 (0.0%)	

**Figure 2:**
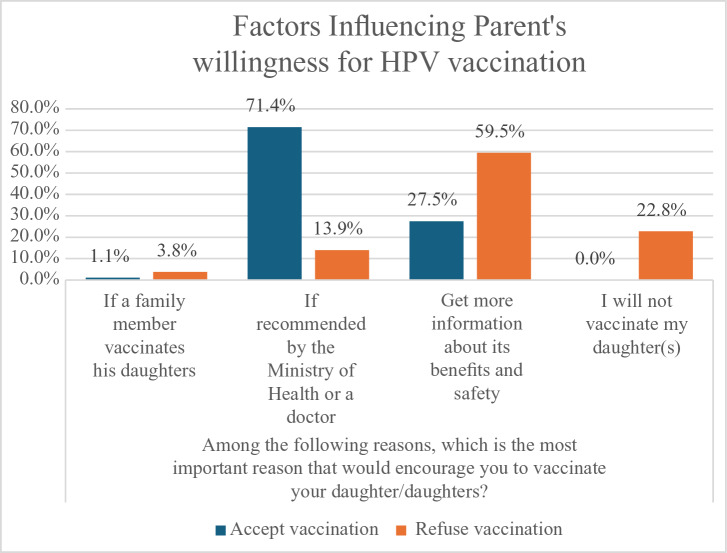
Factors that would encourage parents to have their daughters vaccinated against HPV among those who accepted and refused the vaccine during the school-based program.

Figure 2 shows that 59.5% of participants who refused the vaccine mentioned that they would be encouraged if they received more information on its benefits and safety compared to 27.5% who accepted. Compared to 71.4% of those who accepted the vaccine, only 13.9% of those who refused said they would be encouraged if it was recommended by the Ministry of Health or a doctor. Among both vaccine accepters and refusers, vaccination among family members was not an important encouraging factor (less than 5%). In addition, 22.8% of those who refused the vaccine said they will not vaccinate their daughter(s).

## Discussion

This study examined parental knowledge, vaccine acceptability, and factors influencing the decision-making process regarding HPV vaccination among parents in Rabigh, Saudi Arabia.

We found poor awareness and limited knowledge about HPV infection, despite most parents being familiar with cervical cancer before the campaign. Prior knowledge of HPV, including its clinical presentation and modes of transmission, was limited. Similar knowledge gaps have been reported in other regions of Saudi Arabia, including Riyadh, Jazan, and the Eastern Region, highlighting a widespread lack of awareness among parents [8, 13, 20].

Notably, 98.5% of parents were aware of the association between HPV infection and cervical cancer, while 96.2% recognized the protective role of the HPV vaccine. However, only 42.1% had prior knowledge of HPV before the school-based vaccination campaign, suggesting a substantial increase in awareness likely attributable to the campaign. These findings indicate a higher level of parental knowledge compared to a study in Tabuk conducted during 2022–2023, where only 62.4% of parents of middle-school girls identified HPV as a cause of cervical cancer, and 65.2% acknowledged the vaccine’s preventive role [15]. A similar percentage of parents accepted the vaccine in both studies (30.2% in Rabigh and 34.7% in Tabuk).

Parents with good knowledge were more likely to accept vaccination for their daughters (84.2% versus 67.3%), consistent with a 2022 study in the Western region, where 90% of knowledgeable parents expressed willingness to vaccinate their daughters [21].

The primary reason for vaccine refusal among parents was concern over potential side effects and complications, consistent with findings from a study on Qatari parents [22]. This highlights the critical need for national educational initiatives led by the Ministry of Health to enhance awareness of the HPV vaccine’s safety and benefits. Additionally, healthcare professionals play a pivotal role in addressing parental concerns through evidence-based counselling. Conversely, a separate study in Saudi Arabia identified the perception of daughters being too young and unmarried as the predominant reason for vaccine rejection [15].

Socioeconomic status (SES) was consistently associated with parental decision-making regarding HPV vaccination. Acceptance rates increased with higher educational qualifications and household income and were more prevalent among families where the participating parent was employed. These findings align with previous studies conducted in Saudi Arabia and regionally [18, 20].

Women from lower SES backgrounds are at increased risk of developing cervical cancer, which has been attributed to earlier onset of sexual activity and higher fertility rates in this population. Consequently, lower vaccination uptake in this group may diminish the overall effectiveness of immunization programs in reducing cervical cancer incidence [18, 23].

The Internet was the most frequently utilized source of additional information about the HPV vaccine, aligning with findings from a study in Makkah, where the Internet and social media were the primary sources of HPV infection and vaccination [13]. However, in the present study, when examined separately, vaccine acceptance was significantly higher among parents who relied on Internet searches compared to those who turned to social media, where fewer than half accepted vaccination.

This discrepancy suggests differences in the quality of information accessed through Internet searches versus social media platforms, raising concerns about the spread of anti-vaccine narratives on social media. These findings highlight the need for targeted digital health initiatives to enhance public awareness, dispel misinformation, and increase vaccine acceptance through trusted online resources. The highest vaccine acceptance was observed among parents who obtained information from healthcare providers, reinforcing the indispensable role of medical professionals in shaping public perceptions and promoting evidence-based vaccination decisions.

Vaccine acceptability was higher when both parents participated in the decision-making process, compared to cases where only one parent made the decision. A study in Sharjah found that fathers’ education level increased vaccine acceptance [24]. Further exploration is needed to determine whether incorporating both parents into the vaccination consent process could enhance acceptance rates.

Among parents who refused vaccination, 48.1% cited concerns about side effects and complications as the primary reason. Similarly, when asked about factors that could encourage vaccination, 59.9% indicated that receiving more information about the vaccine’s benefits and safety would influence their decision. These findings highlight the need for comprehensive educational initiatives to address safety concerns and misinformation, thereby improving HPV vaccine uptake.

Most parents who accepted the vaccine cited recommendations from the Ministry of Health or their doctor as the primary influencing factor. These findings are consistent with a study in Riyadh, where recommendations from a trusted physician, knowledge about the vaccine, and government decrees were identified as the most significant determinants in the decision to vaccinate [17], emphasizing the critical role of healthcare professionals and health authorities in advocating for immunization programs.

A national study in 2022 using a validated questionnaire specifically addressing vaccine hesitancy found that 34% parents of adolescent females were hesitant about the HPV vaccine. Besides regional variation, lower household income and lack of recommendation by physicians were associated with higher levels of hesitancy [25].

However, among those who refused vaccination, only 13% considered medical recommendations as a key factor, indicating a distinct difference in decision-making processes between vaccine acceptors and refusers. Notably, 22.8% of parents who declined the vaccine stated that they would not consider vaccinating their daughters, reflecting a strong resistance among a segment of the population. These findings highlight the necessity for tailored and targeted interventions to identify this group and address vaccine hesitancy.

## Strengths and Limitations

While numerous studies have explored parental knowledge of HPV vaccination, this study is among the few that assessed the direct association between knowledge and vaccine acceptance with a focus on a population that has been offered the vaccine. By examining differences between parents who accepted and refused vaccination, this study contributes to filling the existing knowledge gap regarding the impact of awareness on vaccine acceptability. Additionally, distributing the questionnaire through school authorities facilitated a broader and more representative sample, ensuring a more efficient and reliable data collection process. Smaller cities and semi-rural regions such as Rabigh are generally underrepresented in research.

Despite these strengths, the study has some limitations. The internal consistency of the 10-item knowledge scale was moderate, which may introduce some measurement error. Future studies could refine and expand the item pool to improve reliability.

Furthermore, the sample included a higher proportion of mothers, limiting the availability of data on fathers’ employment status and education levels, for about two-thirds of families, which may play a significant role in vaccine acceptance. However, given that parental socioeconomic status is often correlated, the study still identified strong associations between SES indicators and vaccine acceptance. Future research should place greater emphasis on exploring fathers’ roles and perspectives in HPV vaccination decisions. Additionally, 7.5% of eligible parents who started the survey and met the inclusion criteria did not consent to participate. While this proportion is relatively small, it may indicate the sensitive nature of the topic, particularly within a conservative society. If this group differed significantly from those who completed the survey, selection bias may have influenced the findings. Future studies should consider strategies to address potential nonresponse bias and ensure broader participation.

## Conclusion

This study examined the impact of parental knowledge on HPV vaccine acceptance among parents of middle-school girls. Despite limited overall awareness, parents with greater knowledge of the vaccine were significantly more likely to accept it. Higher education levels and family income were also positively associated with increased vaccine acceptance. Additionally, parents who sought information from healthcare professionals or the Internet demonstrated higher acceptance rates. Recommendations from the Ministry of Health and guidance from medical practitioners played a crucial role in shaping parental decisions. Notably, nearly one-quarter of parents who declined vaccination expressed firm resistance, highlighting the importance of addressing underlying beliefs and barriers to achieve vaccination levels that contribute to cervical cancer elimination goals.

Based on these findings, future research should focus on understanding the key factors influencing parental refusal of the HPV vaccine, particularly examining the role of fathers in vaccine decision-making and the influence of social media in disseminating both accurate and misleading information about HPV and its vaccine. Efforts should be directed toward improving awareness and addressing misconceptions among parents through targeted educational interventions. To ensure a comprehensive understanding of vaccine acceptance, future studies should incorporate a more diverse sample across various regions and socioeconomic backgrounds in Saudi Arabia. This approach would provide a more representative perspective on the determinants of HPV vaccine acceptance, enabling the development of effective, evidence-based strategies to enhance vaccination uptake nationwide.

## Data Availability

The data supporting the findings of this study are deposited in Zenodo and are available from the corresponding author upon reasonable request [19].

## References

[r1] Bruni L, Diaz M, Castellsagué X, Ferrer E, Bosch FX, de Sanjosé S. Cervical human papillomavirus prevalence in 5 continents: Meta-analysis of 1 million women with normal cytological findings. J Infect Dis. 2010;202(12):1789–1799. doi:10.1086/657321. 21067372

[r2] Manini I, Montomoli E. Epidemiology and prevention of human papillomavirus. Ann Ig. 2018;30(4 Supple 1):28–32. doi:10.7416/ai.2018.2231. 30062377

[r3] World Health Organization. Cervical cancer. Published 2025. Accessed December 16, 2024. https://www.who.int/news-room/fact-sheets/detail/cervical-cancer.

[r4] Tornesello ML, Cassese R, De Rosa N, et al. High prevalence of human papillomavirus infection in Eastern European and West African women immigrants in South Italy. APMIS. 2011;119(10):701–709. doi:10.1111/j.1600-0463.2011.02784.x. 21917007

[r5] Albayat SS, Mundodan JM, Elmardi K, et al. Knowledge, attitude, and practices regarding human papilloma virus vaccination among physicians in Qatar. Women’s Health (Lond). 2024;20:17455057241227360. doi:10.1177/17455057241227360. 38282514 PMC10826392

[r6] Cui M, Wang Y, Liu Z, et al. The awareness and acceptance of HPV vaccines among parents of primary and junior high school students in China: A meta-analysis. Infect Med (Beijing). 2023;2(4):273–282. doi:10.1016/j.imj.2023.11.003. 38205181 PMC10774669

[r7] World Health Organization. Global Strategy to Accelerate the Elimination of Cervical Cancer as a Public Health Problem. World Health Organization; 2020:56. https://iris.who.int/server/api/core/bitstreams/4e245e89-ddcc-488f-97c7-9de5e08524ef/content.

[r8] Alherz FA, Alamri AA, Aljbreen A, Alwallan N. Knowledge of cervical cancer, human papillomavirus (HPV), and acceptance of the HPV vaccine among parents of daughters in Riyadh, Saudi Arabia. J Infect Public Health. 2024;17(5):789–794. doi:10.1016/j.jiph.2024.03.014. 38520759

[r9] Whitworth HS, Gallagher KE, Howard N, et al. Efficacy and immunogenicity of a single dose of human papillomavirus vaccine compared to no vaccination or standard three and two-dose vaccination regimens: A systematic review of evidence from clinical trials. Vaccine. 2020;38(6):1302–1314. doi:10.1016/j.vaccine.2019.12.017. 31870572

[r10] Saudi Food and Drug Authority. Gardasil 9. Saudi Drugs Information System. Accessed March 20, 2025. https://sdi.sfda.gov.sa/home/Result?drugId=4035&form=MG0AV3&form=MG0AV3.

[r11] Saudi Food and Drug Authority. Cervarix. Saudi Drugs Information System. Accessed March 20, 2025. https://sdi.sfda.gov.sa/Home/Result?drugId=2782.

[r12] Developed and Maintained by Ministry Of Health. Chronic Disease—Cervical Cancer. Accessed November 19, 2023. https://www.moh.gov.sa/en/awarenessplateform/ChronicDisease/Pages/CervicalCancer.aspx.

[r13] Darraj AI, Arishy AM, Alshamakhi AH, et al. Human papillomavirus knowledge and vaccine acceptability in Jazan Province, Saudi Arabia. Vaccines (Basel). 2022;10(8):1337. doi:10.3390/vaccines10081337. 36016225 PMC9413274

[r14] Frazer IH. The HPV vaccine story. ACS Pharmacol Transl Sci. 2019;2(3):210–212. doi:10.1021/acsptsci.9b00032. 32259056 PMC7089001

[r15] Alaamri AM, Alghithi AM, Salih S, Omer HM. Acceptance and associated risk factors of human papillomavirus vaccine among parents of daughters in intermediate schools in Tabuk City, Saudi Arabia. Cureus. 2023;15(8):e43483. doi:10.7759/cureus.43483. 37711956 PMC10499461

[r16] Almatrafi RS, Kamel S, Algarni AD, et al. The impact of an educational program on the awareness and knowledge of human papilloma virus (HPV) vaccine among secondary school girls in Saudi Arabia. Cureus. 2024;16(7):e64957. doi:10.7759/cureus.64957. 39161480 PMC11331014

[r17] Fallatah DI, Khalil MA, Abd ElHafeez S, et al. Factors influencing human papillomavirus vaccine uptake among parents and teachers of schoolgirls in Saudi Arabia: A cross-sectional study. Front Public Health. 2024;12:1403634. doi:10.3389/fpubh.2024.1403634. 39494075 PMC11528711

[r18] Mouallif M, Bowyer HL, Festali S, et al. Cervical cancer and HPV: Awareness and vaccine acceptability among parents in Morocco. Vaccine. 2014;32(3):409–416. doi:10.1016/j.vaccine.2013.10.069. 24188754

[r19] Alghanmi R, Alkhalawi E, Albeladi R, et al. Data of parental knowledge and acceptance of HPV vaccine in Rabigh’s school, Saudi Arabia. Zenodo. July 2025. doi:10.5281/zenodo.15802758. PMC12880011 41659211

[r20] Alnaeem L, Alanizi S, AlQarni G, Alwadani J, Bomouzah F, Ali Z. Acceptance, knowledge, and attitude of parents toward the human papillomavirus vaccine in the eastern region of Saudi Arabia: A cross-sectional study. Cureus. 2023;15(12):e51293. doi:10.7759/cureus.51293. 38283478 PMC10822678

[r21] Alkalash SH, Alshamrani FA, Alhashmi Alamer EH, Alrabi GM, Almazariqi FA, Shaynawy HM. Parents’ knowledge of and attitude toward the human papillomavirus vaccine in the Western Region of Saudi Arabia. Cureus. 2022;14(12):e32679. doi:10.7759/cureus.32679. 36660531 PMC9846376

[r22] Hendaus MA, Hassan M, Alsulaiti M, et al. Parents attitudes toward the human papilloma virus (HPV) vaccine. J Family Med Prim Care. 2021;10(7):2488–2493. doi:10.4103/jfmpc.jfmpc_1122_20. 34568124 PMC8415674

[r23] Yin D, Morris C, Allen M, Cress R, Bates J, Liu L. Does socioeconomic disparity in cancer incidence vary across racial/ethnic groups? Cancer Causes Control. 2010;21(10):1721–1730. doi:10.1007/s10552-010-9601-y. 20567897 PMC2941051

[r24] Saqer A, Ghazal S, Barqawi H, Babi JA, AlKhafaji R, Elmekresh MM. Knowledge and awareness about cervical cancer vaccine (HPV) among parents in Sharjah. Asian Pac J Cancer Prev. 2017;18(5):1237–1241. doi:10.22034/APJCP.2017.18.5.1237. 28610408 PMC5555529

[r25] Othman S, Ghamri R, Alhamadah W, et al. Prevalence and characteristics of HPV vaccine hesitancy among parents of adolescent females across Saudi Arabia. Front Public Health. 2024;12:1501358. doi:10.3389/fpubh.2024.1501358. 39917529 PMC11798979

